# Combining regenerative medicine strategies to provide durable reconstructive options: auricular cartilage tissue engineering

**DOI:** 10.1186/s13287-015-0273-0

**Published:** 2016-01-28

**Authors:** Zita M. Jessop, Muhammad Javed, Iris A. Otto, Emman J. Combellack, Siân Morgan, Corstiaan C. Breugem, Charles W. Archer, Ilyas M. Khan, William C. Lineaweaver, Moshe Kon, Jos Malda, Iain S. Whitaker

**Affiliations:** Reconstructive Surgery & Regenerative Medicine Research Group, Swansea University Medical School, Room 509, ILS2, Swansea, SA2 8SS UK; The Welsh Centre for Burns and Plastic Surgery, Morriston Hospital, Swansea, SA6 6NL UK; Department of Orthopaedics, University Medical Center Utrecht, Utrecht, 3584 CX The Netherlands; Department of Plastic and Reconstructive Surgery, University Medical Center Utrecht, Utrecht, The Netherlands; KhanLab, Swansea University, ILS2, Swansea, SA2 8SS UK; Division of Plastic Surgery, University of Mississippi Medical Center, Jackson, Mississippi 39216 USA; Department of Equine Sciences, Faculty of Veterinary Medicine, Utrecht University, Domplein 29, 3512 JE Utrecht, The Netherlands

## Abstract

Recent advances in regenerative medicine place us in a unique position to improve the quality of engineered tissue. We use auricular cartilage as an exemplar to illustrate how the use of tissue-specific adult stem cells, assembly through additive manufacturing and improved understanding of postnatal tissue maturation will allow us to more accurately replicate native tissue anisotropy. This review highlights the limitations of autologous auricular reconstruction, including donor site morbidity, technical considerations and long-term complications. Current tissue-engineered auricular constructs implanted into immune-competent animal models have been observed to undergo inflammation, fibrosis, foreign body reaction, calcification and degradation. Combining biomimetic regenerative medicine strategies will allow us to improve tissue-engineered auricular cartilage with respect to biochemical composition and functionality, as well as microstructural organization and overall shape. Creating functional and durable tissue has the potential to shift the paradigm in reconstructive surgery by obviating the need for donor sites.

## Introduction

The combined efforts of cell biologists, material scientists, tissue engineers and reconstructive surgeons and associated converging technologies [1] in the 21st century have put us in an enviable position compared with our predecessors. Due to recent advances in regenerative medicine and additive manufacturing we are entering into an age where we have the potential to replace ‘like with like’, by improving the quality of engineered tissue with respect to biochemical composition and functionality, as well as microstructural organization and overall shape.

The importance of regenerative medicine as an emerging discipline is gaining worldwide recognition with a predicted world market value ranging from $2 to 500 billion per annum in 5–10 years [2]; and the assumed finite reservoir of future value has encouraged many countries to invest in this area of research. This value could be exceeded if the barriers to translation and commercialization were overcome. Current research in tissue engineering is geared towards elucidating the appropriate compositional elements (biomaterials, biomolecules and cell sources) as well as methods of assembly. To drive translation of innovative regenerative medicine treatment options to preclinical studies and clinical trials, clinicians need to embed themselves as an essential part of the multidisciplinary team.

One area within the field of plastic and reconstructive surgery that has the potential to benefit from recent advances in regenerative medicine and biomanufacture is auricular reconstructive surgery [3–7]. Abnormal appearance of the ears has a profound effect on self-confidence, quality of life and psychosocial development [8–11], and even minor disfigurement can cause psychological distress. Although the need for total ear reconstruction is relatively rare (e.g. microtia 1–17:10,000 births [12]), partial ear reconstruction owing to acquired defects (trauma, burns or cancer occurs in >1:500 of the population) is more commonly required. Total autologous reconstruction, due to its physical and aesthetic prominence, features in the lay press disproportionately often; and from time to time trumpets standard methods of reconstruction that have been used for decades [13, 14]. At the same time, media coverage of scientific advances often leads patients to believe that three-dimensional printing of auricles lies around the next corner [15–18].

The first tissue-engineered, ear-shaped appendages made from bovine chondrocytes and biocompatible scaffolds by the Vacanti group were prone to deformation when xenografted onto immune-compromised mice, highlighting the lack of long-term stability [6]. We will use the lessons learnt from auricular cartilage tissue engineering to illustrate how combining additive manufacturing and regenerative medicine for tissue-engineering purposes can be used to create functional and durable tissue with potential to shift the paradigm in reconstructive surgery.

## Contemporary autologous auricular reconstruction

The current gold standard of autologous auricular reconstruction is considered one of the most challenging operations in reconstructive surgery largely due to the complex three-dimensional anatomy [19]. The benchmark work by Tanzer, who was the first to describe the use of autologous costal cartilage to create a three-dimensional auricular framework [20–22], has had a significant impact on current strategies. Subsequent work by Brent [23–28], Park [29, 30], Nagata [31–36] and Firmin [37] played a pivotal role in refinement of surgical techniques for ear reconstruction (Table 1). Two-stage total auricular reconstruction is now the standard treatment across the European Union and the United States [12]. The benefits of autologous auricular reconstruction compared with allogenic options such as silastic [38–40] or porous polyethylene (Medpor®; Stryker, Kalmanzoo, MI, USA) [41–43] are high biocompatibility [44], long-term stability, immunocompatibility [5] and the ability to grow with the patient [5, 7, 45].

**Table 1:** Summary of total autologous auricular reconstructive techniques

Surgeon	Technique	Pros	Cons
Tanzer [20–22]	Four stages: 1. Rotation of the lobule into a transverse position 2. Fabrication and placement of a costal cartilage framework 3. Elevation of the ear from the side of the head 4. Construction of a tragus and conchal cavity	– First stepwise total auricular reconstruction – Good results	– Multiple operations – Transposing lobule first poses risk of vascular compromise of skin flap [64]
Brent [23–28]	Four stages: 1. Rib cartilage framework fabrication and placement 2. Lobule transposition 3. Elevation of framework and creation of a retroauricular sulcus 4. Conchal excavation and tragus construction	– Good contour – Postoperative drain limits complications of bolster dressings [64]	– Multiple operations – Lack definition of conchal bowl [64] – Composite skin/cartilage tragal grafts can contract [37]
Nagata [31–36]	Two stages: 1. Fabrication of costal cartilage framework including the tragus, conchal excavation and rotation of the lobule 2. Elevation of framework, placement of cartilage graft in auriculocephalic sulcus, covered with temporoparietal fascial flap and skin graft	– Less operations – High-definition framework to create a good tragus [64]	– More cartilage needed – Detailed framework so long learning curve – Minimum age 10 years – Partial necrosis of posterior flap [37] – Wire sutures increase extrusion [37]

To date, costal cartilage has proven to be the only source of cartilage with an adequate quantity, integrity and acceptable morbidity [46]. The auricular framework usually requires three to four costal cartilage segments, which can be harvested ipsilaterally or contralaterally [21, 47], providing an immunocompatible solution for restoration of the auricle. There have been various advances in the surgical approach to autologous auricular reconstruction; these include the transition towards single-stage procedures [29, 48, 49], and the use of three-dimensional imaging [50, 51] and templates [52] to better match the native ear. The fundamental principles, however, have remained the same since Harold D. Gillies [53] was one of the first to use autologous rib cartilage for auricular reconstruction. The techniques provide consistently excellent results from experienced surgeons but are not without their limitations, creating a clinical need for a tissue-engineered solution.

## Limitations of current auricular reconstructive techniques

### Donor site morbidity

Although there is rich experience worldwide in cartilage harvest for auricular reconstruction, there is a relative paucity of large series investigating donor site morbidity. Uppal et al. [54] reported on 42 patients and found that the most common problems were chest wall pain and clicking, whilst the most serious was a pneumothorax. Other authors have highlighted chest wall contour deformity as a complication, which can be a particular problem when using Nagata’s technique [31–36] that requires the use of a greater amount of cartilage compared with the Brent technique [23–28]. This problem can be minimized by delaying rib cartilage harvest until patients are older (>10 years of age) and leaving the perichondrium intact to allow regeneration of costal cartilage over time [55–59]. Despite these efforts, thoracic computed tomography with three-dimensional reconstruction confirms localized skeletal donor site deformities as late as 6 months after surgery [60]. Hypertrophic scarring is another complication that is particularly common in this group of patients [55, 61–63], although careful placement of surgical incisions can make them less obvious [64]. These surgical adaptations, although useful in reducing donor site morbidity, do not eliminate the problems altogether (Table 2).

**Table 2:** Donor site morbidity associated with total autologous auricular reconstruction

	Donor site morbidity	Incidence	Reference	Total number of patients per study
Early	Pneumothorax	3 (1 %)	[56, 57]	270
		19 (22 %)		88
	Atelectasis	4 (22 %)	[55, 57]	18
		7 (8 %)		88
	Pleural effusion	–	[58]	–
Delayed	Persistent pain	6 (14 %)	[54]	42
	Thoracic scoliosis	4 (25 %)	[55]	16
	Seroma	9 (8 %)	[59]	108, rhinoplasty group
	Clicking	3 (7 %)	[54]	42
	Abnormal scarring	0 (0 %)	[54, 57, 61–63]	42
		3 (2.7 %)		110
		12 (14 %)		88
		14 (5.3 %)		264
		21 (6.5 %)		322
	Contour deformity	3 (7 %)	[54, 55, 57]	42
		16 (50 %)		32
		22 (25 %)		88

### Long-term limitations of the reconstructed auricle

The autologous costal cartilage used in traditional auricular reconstruction can calcify [23, 65] and become resorbed [64, 66, 67] over time. This means that the reconstructed ear may become stiff or thickened [23, 65], with loss of projection or definition [37, 64, 66, 67] resulting in a variable final aesthetic result. The fibro-cartilaginous donor tissue is also intrinsically different in terms of flexibility and strength from the native elastic cartilage it aims to reconstruct [23, 64]. The close association of skin and cartilage in the ear can also render the dermal blood supply vulnerable during dissection and this may cause a relative ischaemia leading to constriction, which can further distort the intended shape [37]. Furthermore, skin flap necrosis or postoperative infections can lead to the rare but serious problem of extrusion [28, 36, 37, 64] (Table 3).

**Table 3:** Long-term limitations of autologous auricular reconstruction

Long-term limitations		Reasons
Stiffness		1. Different biomechanical properties of fibrocartilage donor [23] 2. Heterotopic calcification [23, 65]
Extrusion		1. Skin flap necrosis [37] 2. Wire sutures to assemble cartilage framework [36, 37] 3. Wound infection or pressure dressings [28, 64]
Projection loss		1. Effacement of postauricular sulcus due to contraction of skin grafts [37, 67]
Distortion		1. Constriction of skin and soft tissue overlying the construct due to scarring or ischaemia [37] 2. Cartilage degradation and resorption leading to loss of definition [64, 66, 67]

### Technical considerations

The timing of total autologous auricular reconstruction is determined by the balance between availability of sufficient donor-site costal cartilage, usually adequate by age 6, and avoiding psychosocial problems associated with peer perception when starting school between ages 5 and 6 [19, 21, 68, 69]. The surgical techniques themselves are complex and involve shaping the harvested cartilage to match the contralateral ear either by eye, using image-acquisition technology [50, 51, 70] or via templates [52, 63]. Consistently excellent results require a prolonged period of training to build up the expertise and experience from only a few world experts who have refined their techniques over many years [23–37, 62–64]. This development of expertise will become increasingly difficult with expanding trainee numbers in reconstructive surgery and the potential reduced training time, which will limit the availability of world-class results to the general population.

## Auricular reconstruction combining regenerative medicine and additive manufacturing

Over the past decade there has been an incremental expansion of the applications of tissue-engineering technology to reconstructive surgery. Historically, tissue engineering has involved cell culture techniques, cell seeding of scaffolds to mimic extracellular matrix and growth of tissue in a bioreactor. These approaches have attempted to generate durable auricular cartilage replacements matching the functional and aesthetic properties of normal ears [6, 7, 45, 71–74]. Although progress has been made and techniques have been refined, it is not yet possible to mimic the functional characteristics of native ears (flexibility, strength and elasticity) whilst maintaining the correct shape of the ear after insertion under the skin for prolonged periods of time [6, 75].

**Fig. 1:**
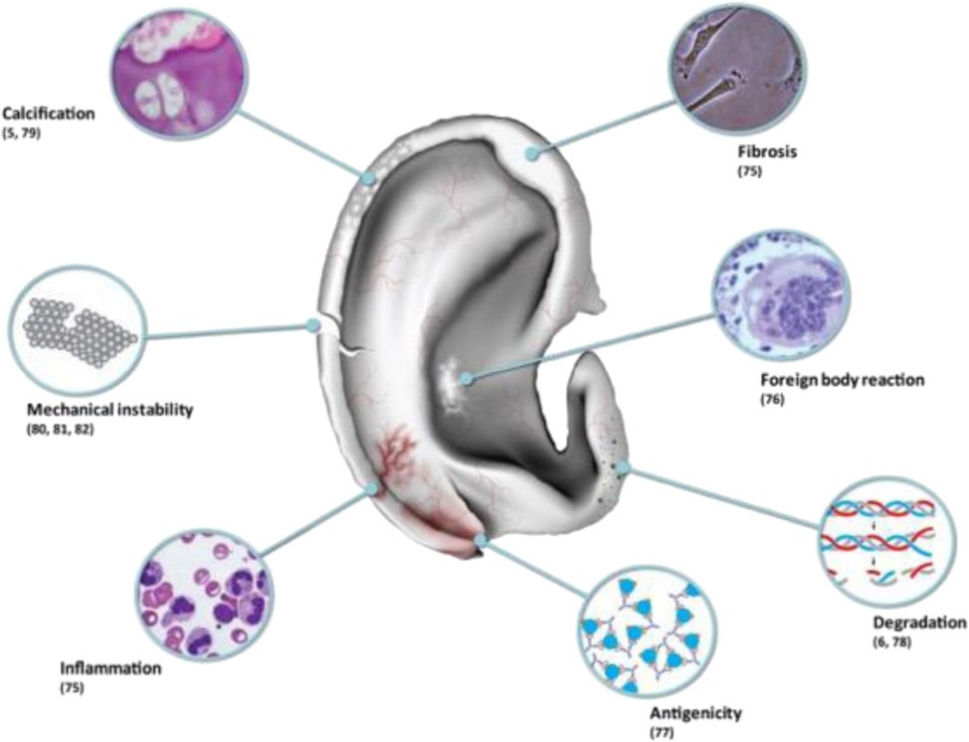
Limitations of current tissue-engineered auricular cartilage constructs

The heterogeneity in approaches indicates that we do not yet have a long-lasting tissue-engineered solution. A number of limitations have been identified with current tissue-engineered auricular cartilage, as outlined in Fig. 1. Constructs implanted into immune-competent animal models have been observed to undergo inflammation, fibrosis [75] and foreign body reaction [76]. This is particularly problematic with polymeric scaffolds, such as poly(lactic acid) or poly(glycolic acid) scaffolds, whose degradation products promote antigenicity [77]. In many cases, the unrelated cell sources produce immature neo-cartilage that is prone to degradation [6, 78], is prone to calcification [5, 79] and is easily breakable [80–82].

Lessons from tracheal tissue engineering indicate that constructs from non-related stem cell sources combined with synthetic scaffolds have their limitations [83–85]. This suggests that simply combining the building blocks for tissue engineering is not sufficient for true regeneration. Observations in developmental biology have shown that function follows form [86], and in order to create true ‘like for like’ tissue for reconstructive surgery it is important to accurately reproduce native tissue anisotropy [87]. In the case of auricular cartilage, the ideal scenario consists of the right compositional elements: such as tissue-specific (auricular cartilage) stem cells, non-immunogenic scaffolds, the correct method of assembly to replicate the native microenvironment and biological induction of maturation using growth factors (Table 4).

**Table 4:** Potential future benefits and challenges of combining regenerative medicine with additive manufacturing

	Feature	Benefits	Challenges
Bioprinting	Control over macrostructure and microstructure of tissue produced	Replicate anatomical form Reduce surgical technique learning curve	Biomechanical properties of bioinks Effect of printing on cells Printing resolution
	Patient-specific macrostructure from image acquisition (CT/MRI)	Reduce variability in surgical outcomes	Macrostructure may alter during bioreactor maturation
	Manufacture ex vivo	Avoid donor site morbidity Reduce operating time	Potential for contamination Regulatory constraints
Regenerative medicine	Tissue-specific stem cells to improve quality and functionality of engineered tissue	True ‘like for like’ replacement Restoring native anisotropy allows improved matching of mechanical properties	Genetic stability and differentiation capacity of cells after prolonged expansion in culture
	Tissue maturation utilizing growth factors	Reduce degradation and constriction	Optimal growth factor combinations and temporal effects

*CT* computed tomography, *MRI* magnetic resonance imaging

Successful translation of tissue engineering for any type of reconstruction will require significant infrastructure and scale-up technology. The most commercially viable and widespread use is likely to come from ‘off the shelf’ tissue-engineered products. Clinical grade processing, scale-out and commercialization all incur substantial time and cost. It is important for clinicians to have a working knowledge of these barriers to translation. These barriers, however, are not unique to tissue engineering; many countries have large translational income streams following successful engagement with large biotech companies with streamlined regulatory processes [88].

### Tissue-specific stem cell utilization

For complex three-dimensional composite tissues such as the ear, it is still unknown how to sufficiently engage and enhance the body’s own repair processes to regenerate lost tissue. The complexity, in this case, must be engineered. To regenerate auricular structures faithfully using extrinsic mechanisms, we need to use biocompatible scaffolds that are populated by auricular chondrocytes which in conductive environments will produce elastic cartilage. In the current paradigm, the cellular component is derived from stem cells that are induced to continually renew and that when directed have the potential to differentiate into chondrocytes and make cartilage. Several groups have used bone marrow-derived stem cells [89–92], adipose-derived stem cells [93–95] and blood-acquired mesenchymal progenitors [96] to various degrees of efficacy. Observations in developmental biology indicate that, for true ‘like for like’ replacement, tissue-specific stem cell sources are needed. Chondroprogenitors were first identified in articular cartilage [97, 98], and more recently it has been shown that they can also be found in the perichondral layer of auricular cartilage [99–101]. Chondroprogenitors from this layer have the power to proliferate for many generations, producing hundreds of millions of progeny from a single founder cell whilst retaining the capacity to differentiate into auricular chondrocytes and make elastic cartilage [100–102]. A further advantage of using auricular-specific stem cells is that they are able to reconstitute both the perichondrium and chondrium, and thus other functions, such as the ability to produce and react to tissue-specific developmental cues in order to regulate normal growth over the lifetime of an implant. Studies show that using highly related cell sources such as articular, costal and naso-septal chondrocytes, unlike those derived from the auricle itself [103], does not result in production of elastin-containing cartilage which has important physiological and biomechanical consequences [79, 104, 105]. For example, costal cartilage, which is the mainstay of autologous auricular reconstruction [19], eventually forms calcified cartilage following its normal developmental pathway [106–108] and, although more common in old age, calcifications can be encountered in young adults as well [106].

### Maturation process

Whilst tissue-specific stem cells ensure the production of auricular cartilage, the neo-cartilaginous structure formed is immature in phenotype. Immature cartilage is highly active metabolically as it grows and develops, and this tissue in a mature organism may be liable to resorption. In the landmark study of auricular tissue engineering by the Vacanti group, tissue-engineered, ear-shaped appendages made from bovine chondrocytes and biocompatible scaffolds were xenografted into immune-compromised mice [6]. The implanted constructs were initially supported by externally fixed stents, and once the stents were removed the shape of the constructs eventually deformed and shrank. This observation highlights often-overlooked features of tissue-engineered implants, the lack of long-term biochemical and biomechanical stability. These experiments also suggest that susceptibility to resorption may be an intrinsic property of neo-cartilage, as well as resulting from the activity of extrinsic factors, for example inflammatory mediators, following implantation. An internal permanent support, such as a coiled wire, although shown to reduce shrinkage of the tissue-engineered auricle in animal models [109], fails to overcome the recognized complications, particularly extrusion, of implanted synthetic materials [38–40, 42, 43, 110].

**Fig. 2:**
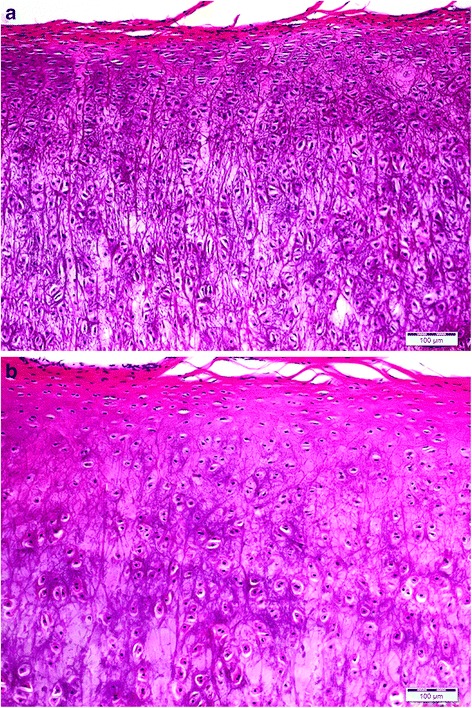
Histology of bovine auricular cartilage (haematoxylin and eosin staining). **a** Immature bovine auricular cartilage demonstrating homogeneous cell organization and high cell density. **b** Mature bovine auricular cartilage showing depth-dependent cell density and organization

The collagen and elastin framework of the native ear can last for an entire lifetime, partly due to extensive chemical cross-linking that over time stabilizes these structural fibres. The process of cross-linking occurs during postnatal maturation providing biochemical heterogeneity [111, 112]. Using the example of articular cartilage, it is a functional adaptation response [78] and in humans may take up to 15 years to become complete [111]. Changes in collagen cross-linking correlate with biomechanical strength of cartilage [113], a recognized yet underused outcome measure of durable tissue-engineered cartilage [5, 114]. The extended time required for maturation and the fact that certain elements of this process require temporally encoded developmental cues may be the root cause for the failure of intrinsic mechanisms of cartilage repair following injury. The lack of maturation is without doubt a major cause of the failure of implanted tissue-engineered cartilage to provide durable replacement tissue for focal lesions in articular cartilage. However, recent work by Khan et al. [115, 116] shows that it may be possible to create implants containing mature cartilage, whose structure and function mimics that of surrounding cartilage, with potential to be resilient to resorption. Their work on native cartilage explants has shown that postnatal maturation can be precociously induced by growth factors fibroblast growth factor (FGF)-2 and transforming growth factor beta-1 (TGF-β1) in immature articular cartilage. Growth factor-treated immature tissue is stiff and smooth and is morphologically as well as biochemically indistinguishable from native adult mature cartilage. There is also evidence that FGF supplementation improves the quality of tissue-engineered elastic neo-cartilage from expanded human auricular chondrocytes [117]. Consequently, a major question that requires our attention is: to what extent does auricular cartilage undergo tissue maturation? Our preliminary work has shown that there are clear morphological differences between mature and immature bovine ears (Fig. 2). By understanding the dynamics of maturational processes in auricular cartilage that underpin these morphological differences, we have the potential to highly accelerate the fabrication of implantable replacement tissues that are both functional and durable.

Nevertheless, the cost-effective production of a scalable solution for craniofacial cartilage replacement tissues, at least in the near term (5–10 years), will require the implantation of allograft material. We are fortunate in this regard that cartilage is immunoprivileged, being avascular, alymphatic and aneural and surrounded by a dense extracellular matrix that is impervious to leukocytes [118, 119]. However, before allogenic constructs become a viable option, human trials are essential to confirm the preliminary in vitro and animal work. Chondrocytes in cartilage rely solely on diffusion to obtain their nutrients and disperse their waste products, and for these reasons it is likely that fabricated implants which are stabilized through growth factor-induced maturation prior to implantation will last the lifetime of the patient.

### Three-dimensional bioprinting

Three-dimensional printing creates objects from a digital model in a layer-by-layer fashion. As such, it offers full control over internal and external architecture of the object, in contrast to subtractive manufacturing approaches. Three-dimensional printing is dramatically altering the way we perceive manufacturing and is an important driving force of the paradigm shift towards digital manufacturing that is now often regarded as the third industrial revolution [120]. In reconstructive surgery, this technology already enables fabrication of patient-specific models for preoperative planning (e.g. autologous free flap reconstruction [121]), patient or surgeon education [122] or intraoperative use (e.g. mandibular [123] or breast [124] reconstruction). Moreover, customized implants to restore anatomical features have also been produced, including partial or complete mandibular replacements [125, 126] and cranial constructs [127, 128].

**Fig. 3:**
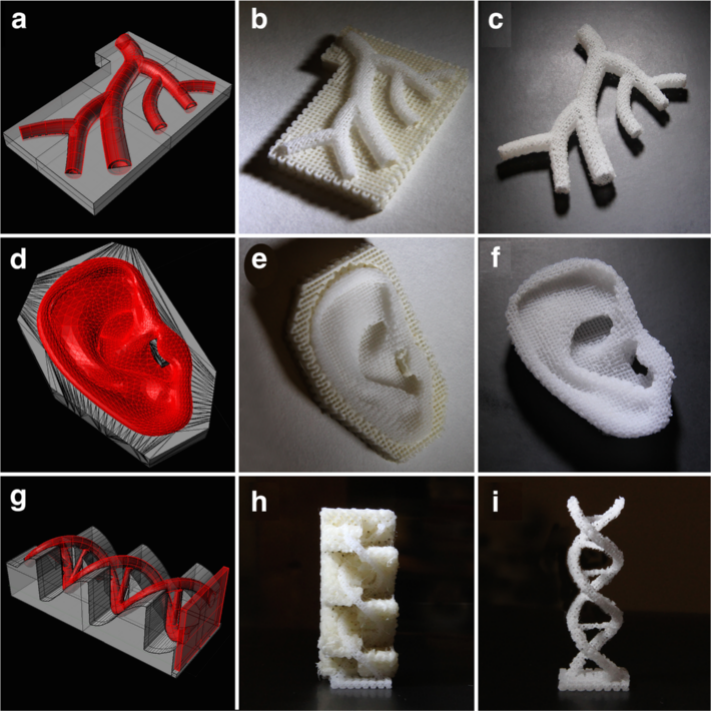
Three-dimensional-printed complex anatomical structures based on polycaprolactone (PCL) with polyvinyl alcohol (PVA) support. **a**–**c** Vascular tree. **d**–**f** Right ear. **g**–**i** DNA helix. **a**, **d**, **g** Computer-aided designs showing permanent (*red*) and sacrificial (*grey*) components. **b**, **e**, **h** Printed structures showing PCL (*bright white*) and PVA (*off-white*). **d**, **f**, **i** PCL scaffold after sacrificing PVA support. Reproduced with permission from Visser et al. [131] and Institute of Physics Publishing

Degradable implants with overhang geometries and internal cavities can be created by the inclusion of temporal support structures from polymers [129–131] (Fig. 3). These scaffolds can then be inoculated with cells. On the other hand, three-dimensional printing can also yield patient-specific moulds in which cell-containing hydrogels can subsequently be cast to obtain implants with complex shapes; for example, for potential restoration of auricular deformities [73, 132]. Nevertheless, these approaches only yield relatively homogeneous constructs and lack the control over specific placement in three dimensions of different biomaterials, cells or other bioactive components, such as growth factors to stimulate specific cellular differentiation.

Three-dimensional bioprinting entails the creation of biological structures for tissue engineering, pharmacokinetic or basic cell biology studies (including disease models) by a computer-aided transfer process for patterning and assembling living and non-living materials with a prescribed three-dimensional organization [133–135]. Hydrogels are widely used as bioinks (i.e. printable biological materials), since they can recapitulate a number of features of the native extracellular matrix and allow cell encapsulation in a highly hydrated three-dimensional environment [136–139]. Biofabrication, using robotic dispensing for example, imposes opposing requirements on the hydrogel materials [51, 133, 140, 141]. Biofabrication of complex structures, such as the auricle, requires a stiff hydrogel for high resolution and mechanical stability on implantation whilst being soft enough to allow cellular migration, proliferation and differentiation. Co-depositing thermoplastic polymer fibres and cell-laden hydrogels can be used to reinforce and hence tailor the mechanical properties of the constructs [138, 142], offering the opportunity to support the long-term maintenance of the implant shape. A more biological approach using in vitro maturation, as already described, could increase construct stiffness via specific tissue matrix deposition [115, 116], although high cell concentrations and a reasonable pre-culturing period would be required. The future advantage offered by three-dimensional bioprinting is biofabrication of more complex structures such as auricular cartilage with an overlying skin and soft tissue envelope due to multiple ‘bioinks’ containing differing cell and hydrogel types.

## Conclusion

Auricular reconstruction is an ideal example of how refinement in surgical techniques over many years can give excellent results in expert hands. However, as with any autologous technique, auricular reconstruction is limited by donor site morbidity. Recent advances in bioengineering and collaborations between stem cell biologists, engineers and clinicians have developed a landscape which provides the opportunity to engineer auricular cartilage constructs that resemble the human ear in shape, size and flexibility.

There are fundamental scientific questions that need to be addressed in order to overcome the current limitations of tissue-engineered constructs for long-term sustainability, including optimizing utilization of tissue-specific stem cells and manipulation of maturation. We have used the auricle as an exemplar to illustrate how combining regenerative medicine with three-dimensional bioprinting has the potential to create custom-made tissue-engineered solutions which obviates the need for a donor site, representing a paradigm shift in reconstructive surgery. It is, however, important to recognize that there are a number of barriers to successful translation which need to be overcome before tissue-engineered products become a commercially viable and widespread alternative to autologous reconstruction.

## Abbreviations

FGF: Fibroblast growth factor
TGF-β1: Transforming growth factor beta-1
